# Simulation training for holistic and systematic health needs assessments of older adults: a qualitative study

**DOI:** 10.1186/s41077-025-00388-6

**Published:** 2025-12-02

**Authors:** Bente Hamre Larsen, Dagrunn Nåden Dyrstad, Helle K. Falkenberg, Peter Dieckmann, Marianne Storm

**Affiliations:** 1 Department of Public Health, Faculty of Health Sciences, University of Stavanger, Postbox 8600 FORUS, 4036 Stavanger, Norway; 2 Department of Quality and Health Technology, Faculty of Health Sciences, University of Stavanger, Stavanger, Norway; 3 National Centre for Optics, Vision and Eye Care, Faculty of Health and Social Sciences, University of South-Eastern Norway, Kongsberg, Norway; 4 USN Research Group of Older Peoples’ Health, University of South-Eastern Norway, Drammen, Norway; 5 Copenhagen Academy for Medical Education and Simulation (CAMES), Center for Human Resources and Education, Capital Region of Denmark, Denmark and University of Stavanger, Copenhagen, Norway; 6 Department of Public Health, Copenhagen University, Copenhagen, Denmark; 7 Faculty of Health Sciences and Social Care, Molde University College, Molde, Norway

**Keywords:** Simulation training, Health care professionals, Health needs assessment, Assessment tools, Older adults, Home care, Home living, Interprofessional collaboration, Health-promotion

## Abstract

**Background:**

As more people age, healthcare professionals require skills in using tools for interprofessional, holistic health needs assessments to support aging in place. While simulation training is recognized to build professional skills, its application in training interprofessional teams by using tools to holistically assess and plan care for older adults at home remains limited.

**Aim:**

To explore healthcare professionals’ perceptions of interprofessional simulation training in assessing the holistic health needs of older adults living at home (i.e., physical, cognitive, mental, sensory, behavioral, and social) and their views on appropriate measures.

**Method:**

A qualitative, exploratory study with five simulation training sessions focused on assessing health needs in older adults living at home. The simulation included 11 participants (nurses, physical therapists, and occupational therapists). The simulation sessions comprised introduction, briefing, scenario with role play, and debriefing, and were conducted in a home-like laboratory. The introduction prepared participants through e-modules. The briefing covered information about the scenario, participant roles, and tools to assess physical, cognitive, mental, sensory, behavioral, and social health needs. Participants chose either an active or an observer role in a scenario involving a health needs assessment in an older adult’s home. This was followed by debriefing during which participants shared their experiences. The debriefing transcripts served as the study data and were analyzed using thematic analysis.

**Results:**

Participants reported that the tools to assess health needs provided systematic and holistic insight on the health of a simulated older adult. They perceived that interprofessional collaboration supported both the assessment process and engagement with the older adult. Participants perceived that assessment scores informed decisions about necessary measures and could enhance older adults’ awareness of their functional abilities, potentially stimulating health-promoting actions. Participants perceived the simulation training as useful and realistic, and both the active and observer roles gave valuable experiences.

**Conclusion:**

Interprofessional simulation training enabled healthcare professionals to practice holistic assessment and identify the health needs of older adult. They perceived that such assessments could inform appropriate measures and promote health. The participants reported the simulation training to be authentic and meaningful.

## Introduction

The World Health Organization (WHO) emphasizes the need to educate the healthcare workforce to support older adults in aging at home [1, 2]. Integrated Care for Older People (ICOPE) is a guide for person-centered assessment and pathways in primary care that focuses on holistic health needs rather than diseases. ICOPE provides tools to detect early declines in intrinsic capacity and the abilities essential for daily functioning [3–5]. These abilities reflect a person’s overall health functions and needs across physical, cognitive, mental, sensory, behavioral, and social domains [3, 6, 7]. Early systematic health needs assessment can identify unmet needs, guide personalized care, and inform timely health-promotion measures that foster independence for aging at home [3, 4, 6, 8–21]. Furthermore, an interprofessional approach to health needs assessment that integrates distinct and overlapping professional skills (e.g., physiotherapy, occupational therapy, nursing) can support the identification and management of health needs [17, 22].

Despite the availability of assessment tools, practices in community settings such as home healthcare remain inconsistent, with variability in both tools used and the health domains assessed [16, 17, 23–29]. Further refinement and digitalization of assessment methods are suggested to support more standardized and holistic health needs assessments [29]. There is also a persistent gap between expected and actual competence levels among healthcare professionals performing assessments [29–33]. A report to the Norwegian Directorate of Health indicated that only 32% of municipalities have a competence plan for professionals assessing health needs [34]. Targeted training is essential to enable interprofessional and systematic assessments of older adults living at home, addressing physical, cognitive, mental, sensory, behavioral, and social health needs [35–37] which supports timely health-promoting measures and ongoing monitoring [29].

A recent scoping review by Larsen et al. suggested that combining active learner participation with teacher-led approaches enhances healthcare professionals’ confidence and competence in assessing health needs and planning care for older adults living at home [19]. Simulation training can integrate both approaches and can support continuous professional development [38]. Simulation fosters interaction between peers and may involve simulators or technical devices [39] and can serve as an effective method for enhancing both technical and non-technical skills in healthcare professionals [40]. Simulation can be defined as a technique to replace or enhance real experience through guided, often immersive, experiences designed to evoke or replicate key aspects of the real world in a fully interactive manner [41]. By replicating real-life scenarios and actively engaging learners, simulation training prepares healthcare professionals to develop skills and adopt new practices [42]. Role-play is a central component of simulation training in healthcare. It allows learners to engage either as active participants in scenario or as observers [43]. Situated within the constructivist paradigm of sociocultural learning theory [44, 45], roleplaying enables participants to construct knowledge individually and collaboratively in a safe environment [46–51]. According to Kolb’s experiential learning model, simulation fosters a cyclical process in which learners engage in scenarios, reflect on their experiences, conceptualize new knowledge, and apply it [52–54].

In the elder care context, simulation has been valued by learners [55] and is considered most effective when combined with traditional teaching methods such as lectures [55, 56]. Simulation training has been shown to strengthen healthcare professionals’ insight and empathy toward older adults [57]. It improves their skills, knowledge, self-efficacy, and communication [56, 58]. It further enhances critical thinking [58], sensory assessment skills [59], advanced care planning [60] and patient education [61].

Although simulation training supports professional development in elder care, it has not been applied to train interprofessional teams to holistically assess the health needs of older adults in their homes [19]. This study aims to explore healthcare professionals’ perceptions of interprofessional simulation training in assessing the holistic health needs of older adults living at home (i.e., physical, cognitive, mental, sensory, behavioral, and social) and their views on appropriate measures. The research question is as follows: How do healthcare professionals reflect on and experience interprofessional simulation training involving the systematic assessment of an older adult’s holistic health needs, and their consideration of appropriate measures based on the assessment?

## Method

### Study design

A qualitative, exploratory design was utilized in this study [62]. Five interprofessional simulation training sessions were carried out, focusing on health needs assessments in older adults. These sessions were conducted in a home-like laboratory setting between October 2022 and January 2023, utilizing role-play scenarios. According to Dieckmann et al., this format allows learners to participate either as active role-players or as observers [43]. To ensure active and consistent role enactment, detailed role descriptions were provided [43, 63, 64], along with a collaboration script to engage observers [65, 66].

### Study participants

Purposive sampling [62, 67] was used to recruit healthcare professionals from municipal health and welfare services whose responsibilities include assessing home-based health needs (e.g., home care, personal assistance) from applications and issuing formal decisions [28]. Eleven participants were recruited from the four Health and Welfare Offices in Stavanger municipality, with two or three participants from each office. Four of them were nurses, three were physical therapists, and four were occupational therapists. All participants had held their positions for at least 2 years and possessed several years of experience in different roles within eldercare.

Among the 11 participants, none had previous full-scale simulation training, though all were familiar with role-play exercises. Each participant took part in two of five half-day simulation sessions. To allow sufficient time in the scenario for the participants to practice using all the tools, the simulation training was carried out in two sessions. Table 1 provides an overview of the participants and simulation session content.

**Table 1:** Simulation session participation and training components

	**Simulation session 1**	**Simulation session 2**	**Simulation session 3**	**Simulation session 4**	**Simulation session 5**
Number of participants	7	4	2	5	4
Professions	3 nurses 2 physiotherapists 2 occupational therapists	1 nurse 1 physiotherapist 2 occupational therapists	0 nurse 1 physiotherapist 1 occupational therapist	3 nurses 1 physiotherapist 1 occupational therapist	1 nurse 1 physiotherapist 2 occupational therapists
Gender	7 female 0 male	3 female 1 male	2 female 0 male	5 female 0 male	3 female 1 male
Health needs assessment content	Physical, cognitive, mental, social, and behavioral	Physical, cognitive, mental, social, and behavioral	Physical and sensory	Physical and sensory	Physical and sensory

### Simulation training

For this study, simulation training was developed and delivered based on Dieckmann’s conceptualization of simulation as a goal-oriented pedagogical approach and a social practice comprising interconnected phases: introduction, briefing, scenario, and debriefing [39, 40].

### Introduction

Participants were encouraged to watch the pre-recorded e-modules prior to the simulation training. These e-modules addressed holistic health needs (physical, cognitive, mental, sensory, behavioral, and social) in old age, health-promoting measures for older adults at home, the importance and benefits of interprofessional collaboration in health needs assessment, skills training, and key elements of simulation training.

#### Briefing

Each simulation session began with a briefing, facilitated by BHL, to familiarize participants with the tools for assessing the holistic health needs of older adults at home. The tools, implemented on an iPad (Apple Inc.), covered physical, cognitive, mental, sensory, behavioral, and social aspects, and included questions, practical tests, and scoring procedures.

Participants then received the scenario descriptions, role instructions, and necessary equipment, and selected their preferred role: one of two healthcare professionals, an older adult, a next of kin, or an observer. The two healthcare professionals had distinct professions and divided the assessment tasks between them for the simulation scenario. The briefing typically lasted 35 min but was extended to 60 min when the vision assessment tool was introduced, as it included additional practical tests that required explanation.

#### Scenario

The scenario depicted role-playing a health needs assessment in an older adult’s home and involved the roles of older adults, next of kin, and healthcare professionals. The simulation was conducted in a home-like laboratory setting, including a kitchen, bedroom, living room, and observation area. Participants engaged with the scenario using structured role descriptions, health needs assessment tools, and a collaboration script [65, 66] to guide interactions and task completion. The collaboration script was used to activate the observers and to increase their learning. Table 2 provides an overview of the scenario, assessment tools, role descriptions, and collaboration script.

**Table 2:** Key components of the simulation training, health needs assessment tools, role description, and collaboration script

Simulation scenario presented for the participants	Ruth, an 80-year-old widow and formerly independent woman, was healthy until her husband’s passing 6 months ago. After a fall at home leading to a 2-day hospitalization and 14 days of rehabilitation, she now feels weaker and less secure and has requested a personal alarm and cleaning assistance. Two municipal healthcare professionals visit Ruth at home with her daughter present, collaborating interprofessional to systematically and holistically assess Ruth’s health needs from a health-promoting perspective
Health needs assessment tools used in the scenario	Physical function: • Short Physical Performance Battery (SPPB) to assess lower extremity function [68, 69] • Clinical Frailty Scale (CFS) to assess frailty [70, 71] Sensory function: • Combined serious sensory impairment to assess hearing [72, 73] • Rehabilitation of sight after stroke to assess vision [74, 75] Cognitive function: • Mini-Cog to assess cognitive impairment [76, 77] Mental function: • Geriatric Depression Scale to assess depression [78] Social function: • 3-item Loneliness scale (UCLA) to assess loneliness [79–81] Behavioral function: • Alcohol Use Disorders Identification Test (AUDIT-4) to assess alcohol use [82, 83] Activity of daily living (ADL): • IPLOS assessment form (The Norwegian Information System for the Nursing and Care Sector) to assess ADL and assistance needs, including housekeeping, personal hygiene, meal preparation, eating, communication, support from next of kin, and perceived safety [84–86]
Role descriptions	Older adult: Ruth misses her husband and depends on her daughter for help. She walks slowly with unsteadiness and has limited social interactions, mild cognitive decline, and difficulty getting out of bed. She eats and drinks little and has back pain, poor vision, and hearing. She struggles with housekeeping, feels insecure and lonely, fears falling, sleeps poorly, and experiences sadness Equipment: Shawl, blanket, glasses (to mimic reduced vision)
	Next of kin: Ruth’s daughter feels overwhelmed and tired. She is worried about mother’s health and declining functionality since father’s passing
	Healthcare professionals: Municipal healthcare professionals collaborate to conduct a health needs assessment using the tools on an iPad. Based on the results, they discuss and recommend health-promoting measures Equipment: iPad with assessment tools, printed assessment forms, vision board, reading test, Mini-Cog clock-drawing test, tape measure, and stopwatch
Collaboration script for observers	During the scenario, please observe the following: Collaboration and communication between healthcare professionals: • How do they collaborate and communicate during assessment? • How is the assessment carried out? Collection of information using tools: • What is effective, and what are the challenges? Interaction and communication with the older adult during the assessment: • How is the older adult informed and reassured during the assessment? • Documentation? Summarizing the assessment? • What measures are offered, and how? General areas for improvement: • Do you see anything that your colleagues could improve?

### Data collection

#### Debriefing

The debriefing consisted of structured reflections [40, 87] and was facilitated by DND. Audio recordings from five debriefings served as the data for the study. Each debrief lasted 30–40 min and was guided by seven key questions based on Gibbs’ reflective cycle [88] (Table 3). Participants talked about and reflected on their experiences with the different roles in the scenario, interprofessional collaboration, the assessment process, the use of tools, the implementation of health-promoting measures, and the overall training experience. Each session concluded with a summary of key learning points, providing closure and reinforcing the take-home messages from the simulation.

**Table 3:** Debriefing questions

Debriefing questions	
Begin by allowing everyone to express their immediate feelings and reactions	
Review the learning objectives Questions: How was the experience of participating in the simulation? How did the older adult and next of kin perceive the assessment? How was the interprofessional collaboration during the assessment? How was the use of assessment tools on iPad and documenting the scores? How was it to consider potential health-promoting measures for the older adult? What feedback does the observers have from the scenario and based on the questions on their collaboration script? How has the overall training experience been?	

### Data analysis

The audio recordings from the debriefings were transcribed into 30 pages of text transcripts which were analyzed using Braun and Clarke’s reflexive thematic analysis [89, 90]. The analysis was inductive, with patterns and themes emerging directly from participants’ experiences to ensure data-driven findings [89, 90]. All authors participated in the data analysis process to develop a shared understanding of the data. The transcripts were coded into segments relevant to the research question, which were then collated into themes. The results section was drafted and revised iteratively to ensure alignment with both the transcripts and the research question. Table 4 presents the phases of the reflexive thematic analysis and the corresponding actions.

**Table 4:** Phases of reflexive thematic analysis

Phases of reflexive thematic analysis	Actions in the analysis phases
Familiarization with the data	BHL transcribed all simulation audio recordings (30 pages). BHL read and re-read the material to gain a deep understanding of the data. MS read all transcripts in full, while the other authors each reviewed transcripts from two debriefing sessions. At the first analysis meeting, BHL presented preliminary patterns and potential initial codes identified across the five debriefing sessions, while all authors contributed their interpretations of the content. Through reflection and discussion, a shared understanding of the dataset was developed
Generating initial codes	Following the first meeting and the development of a shared understanding of the dataset, BHL systematically coded the transcribed material into segments that reflected content relevant to the research questions. NVivo was used in this process [91]. The codes were subsequently discussed and refined in collaboration with MS
Searching for themes	The codes were then examined for patterns and collated into potential themes. In during the second analysis meeting, all authors reviewed and discussed potential themes by examining the grouped codes. A visual map illustrating the relationships between codes and themes was used to support this discussion
Reviewing themes	Potential themes were elaborated through fuller written descriptions, with their content refined through iterative writing and commentary. Each theme was checked against the transcripts and considered in relation to the research questions to ensure accuracy and relevance
Defining and naming themes	Each theme was defined to capture its core essence and meaning and named to reflect its role in the overall analysis and alignment with the research question. All authors contributed ideas to the development of the text
Reporting the findings	The results were drafted and refined through iterative review and revision by all authors

### Ethics

The study was assessed by SIKT—the Norwegian Agency for Shared Services in Education and Research on September 23, 2022. SIKT oversees that Norwegian research projects are carried out in compliance with data protection and privacy requirements in accordance with the General Data Protection Regulation (GDPR), ethical guidelines, and Norwegian legislation [92, 93]. Healthcare professionals in health and welfare offices were invited by their leaders to participate in the study. They received written and verbal information about the project before providing their written informed consent.

## Results

The researchers identified four overarching themes from the reflexive thematic analysis: (1) valuing systematic holistic health needs assessment; (2) enabling feasible and flexible assessment by interprofessional collaboration; (3) tailoring measures based on assessment scores; and (4) learning by engaging in an authentic scenario. The analytic steps from the quotes to the final themes are detailed in Table 5.

**Table 5:** Overview of the reflexive thematic analysis [89, 90]

Codes (in bold) that capture relevant meanings in the dataset pertinent to the research question	Initial themes across the dataset	Refined themes	Final themes
**Information and structure**: “*We do obtain information that we normally wouldn’t get from a regular assessment conversation in current practice. It provides very comprehensive information*”—Participant 2 and 3, simulation 5	The tools provided consistency and structure in the assessment process. Led to holistic information about older adults’ health needs	Using assessment tools led to systematic and holistic health needs assessment	Valuing systematic holistic health needs assessment
**Holistic assessment**: “*Iplos questions and observations of older adults can probably be just as good as SPPB. I use observation a lot, when they show us around the house, *etc.” —Participant 2, simulation 1	Teams reflected about the need to use tools, and interconnectedness of health needs. The assessment was perceived time-consuming, but manageable	Holistic health needs assessment is important and feasible, but still time-consuming	
**Interprofessional collaboration**: “*It felt like there was a lot to keep track of: talking to the older adult, staying focused on the tests, and remembering what I was supposed to do. For example, I forgot to test one eye – it just became too much with the testing and recording results on the iPad. It was helpful to be two healthcare professionals collaborating*”—Participant 1, simulation 3	Interprofessional collaboration resulted in a manageable health needs assessment The collaboration enabled discussions from diverse perspectives	Interprofessional collaboration was vital for holistic health needs assessment	Enabling feasible and flexible assessment by interprofessional collaboration
**Involvement of older adult and next of kin**: “*I felt well cared for because the healthcare professional sat in front of me, maintained eye contact, and addressed me directly, not through my next of kin*”—Participant 1, simulation 4	The interprofessional teams managed natural dialogue with older adults and next of kin during the health needs assessment Older adults felt cared for and respected	Interprofessional collaboration enabled natural dialogue with older adults and next of kin	
**Health-promotion measures**: “*You get a measurable number, a score. The patient becomes more aware of several areas of their own life, for example that they have good balance and can manage a bit more. It is easy to measure whether they decline or improve*”—Participant 3, simulation 1	Assessment scores provided measurable results and enabled tracking over time Scores were translated into health-promoting advice and measures Uncertainty to some areas	Assessment results were used to suggest appropriate health-promotion measures and advice to older adults in the simulation	Tailoring measures based on assessment scores
**Having roles in simulation**: “*The setting is recognizable, making it easy to imagine it as a real situation*”—Participant 1, simulation 3	Valuable to have a role as well as observe The scenario was authentic and engaging	The authentic scenario actively engaged participants in the simulation training	Learning by engaging in an authentic scenario
**Training experience**: “*I will retain what we learned better than if I had only read about it or watched it on a screen*”—Participant 1, simulation 4 “*If we had approached an older adult and tried this for the first time, it would have been a bit like… but now we have experience and a sense of what it can be like… It was great to have the opportunity to practice*”—Participant 4, simulation 2	Pleasantly surprised by the simulation training, which provided valuable learning	Participating in simulations was useful and valuable for learning	

### Valuing systematic holistic health needs assessment

The simulation training allowed interprofessional teams to practice systematic assessments of health needs (i.e., physical, cognitive, mental, sensory, behavioral, and social). During the debriefings, participants noted that the assessment and use of tools offered a holistic view of the older adult’s health needs. They also reported that using the assessment tools addressed areas often overlooked in current practice, such as alcohol use, depression, and vision. Participants said that the tools provided a clear structure with predefined questions, which helped keep the assessment focused and consistent. One participant said:

“*I found the assessment tools on the iPad to be both effective and user-friendly. It is easy to identify any omissions, as these are marked in red. The tools help maintain structure; however, there is something about having to follow a predefined sequence.*”—Participant 1, simulation 1

During debriefing, some participants highlighted the interconnectedness of health needs. One participant commented on how vision impairments and alcohol use can interact to increase fall risk, emphasizing the importance of holistic health needs assessment. However, there were participants who questioned the need to use all the tools for every assessment. One participant noted that open assessment conversations, as practiced in current care, can provide valuable insights into health needs by allowing older adults to share life stories and personal priorities.

Participants said that the number of questions and tests made the assessment time-consuming. The brief screening tools for cognitive function, alcohol use, and loneliness were considered easy to use, whereas the tools for assessing physical function, depression, vision, and hearing were described as more detailed and demanding to administer. While they expressed a preference for concise and straightforward tools, they still found all tools usable and anticipated increased confidence and competence with continued practice.

### Enabling feasible and flexible assessment by interprofessional collaboration

In debriefing, participants who had played roles as healthcare professionals in the scenario talked about how they collaborated when carrying out health needs assessment tools. They said that they had agreed in advance on the distribution of responsibilities for performing the assessment. They reported that prior familiarity with each other’s roles and expertise contributed to a natural collaborative process. The participants emphasized the need for two assessors, as managing the assessment, documenting findings, and attending to both the older adult and the next of kin were too demanding for one person. It was expressed that collaboration made the assessment more manageable, facilitated smoother conversations, and helped avoid awkward pauses. They also reflected on the value of considering older adults’ needs from an interprofessional perspective, as competence and perspectives varied between nurses, physiotherapists, and occupational therapists. However, they noted that such collaboration was rare in their current practice due to time and resource constraints.

There were participants who expressed concerns that the use of assessment tools might create distance between assessors and older adults and limit open-ended dialogue. However, observers noted that the interprofessional teams made the assessment feel natural by adapting the tool’s questions into everyday language, asking follow-up questions when appropriate, and engaging in small talk with the simulated older adult. The participants in role as older adult and next of kin reported feeling safe, cared for, and respected, with attention focused on the older adult. One simulated older adult remarked:

“*No, the systematic assessment did not create any distance for me. Furthermore, it was an advantage that there were two healthcare professionals; they complemented each other*”—Participant 5, simulation 1

Participants suggested adding a standard introduction consisting of a brief overview of the forthcoming assessment, followed by the question “What matters to you?” to encourage more openness regarding patients’ own wishes and reflections.

### Tailoring measures based on assessment scores

During the simulation, healthcare professionals continuously recorded the assessment scores on the iPad. During the debriefing, those in healthcare professional roles noted that overall documentation was manageable, although scoring the vision, physical function, and depression assessments required additional time and effort. They also noted the limited space in the iPad assessment tools for recording supplementary details and suggested using pen-and-paper notes as an additional record. They anticipated that documentation would improve as they became more familiar with the tools.

In the debriefing, the participants talked about that the scores provided measurable outcomes and helped identify risk areas, enabled tracking of changes in areas of health over time, and informed them of whether measures were necessary. However, there were participants expressing some uncertainty about providing advice and recommending measures in areas where they felt less experienced, such as in the sensory, mental, and behavioral domains. They also emphasized the need to develop their knowledge and competencies in these areas. As one participant said:

“*Maybe it’s us as healthcare professionals who need updated information and increased awareness about these domains—for example, vision and the problems it can cause—so that we can provide good advice in this area, not just ask questions and perform tests.*”—Participant 1, simulation 3

During the debriefing, participants reflected that the assessment scores could enhance older adults’ awareness of their abilities and limitations, motivate self-care, and encourage daily physical activity. There were participants emphasizing that, even without specific interventions, identifying and addressing early signs of functional decline can raise awareness, prompt reflection, and motivate health-promoting behaviors in older adults, such as resuming activities like grocery shopping or walking. Participants also highlighted the importance of clearly explaining available health-promotion measures, particularly for older adults who may be unaware of existing support and resources.

### Learning by engaging in an authentic scenario

During the debriefing, participants reflected on their experiences with the simulation training. They stated that they appreciated the opportunity to test the assessment tools in a simulated setting, particularly given their initial unfamiliarity with most tools and with a systematic approach to assessing health needs. Participants emphasized that the training was useful, relevant, and meaningful, noting that it provided a sense of security before conducting such assessments in real-world practice. Although they expressed feeling nervous and uncertain beforehand, they were pleasantly surprised by the experience and regarded it as a memorable learning opportunity. Participants also highlighted that the small-group setting helped to alleviate their initial nervousness.

Participants discussed the option to choose between taking on an active role or observing during the debriefing phase and noted that they appreciated this flexibility, particularly those who found the active role challenging or unnatural. The participants who acted as observers reported that this allowed them to reflect and learn from a distance, while participants in active roles generally did not find this problematic. Some considered the healthcare professional role the easiest to perform due to their familiarity with the assessment tasks. One simulated healthcare professional remarked:

“*I noticed I changed my voice and tone, adapting it to the older adult by speaking loudly and clearly*”—Participant 1, simulation 3

During the debriefing, the participants noted that the simulation training was realistic and authentic, as the laboratory environment resembled a home, and the scenarios closely mirrored real-world assessments. The participants acting as observers described participants in active roles as engaged and realistic, and noted that the setting resembled a home where health needs assessment was conducted. At the same time, some participants reflected that assessments in practice might differ, for example, with next of kin being more actively involved, professionals providing more information to the older adult, and additional small talk occurring beforehand.

## Discussion

This study offers insights into the use of simulation training to prepare interprofessional teams for conducting holistic and systematic health needs assessments of older adults living at home. The results showed that, during the simulation, participants reported having gained insight into using various assessment tools and their practical application, as well as experience with simulation training as a pedagogical approach. Interprofessional teamwork facilitated the assessment process, supported natural dialogue with the simulated older adult, and created opportunities to discuss their health needs and suggest interventions.

During our simulations, participants collaborated in interprofessional teams and applied established assessment tools such as the Physical Performance Battery, Clinical Frailty Scale, Mini-Cog, and Geriatric Depression Scale to carry out holistic health needs assessments. This gave participants a concrete experience of tool use in practice, corresponding to the first phase of Kolb’s learning cycle. This phase fosters skill development and provides the foundation for reflection and deeper learning in subsequent stages [53]. The use of simulation training to enhance healthcare professionals’ skills in assessing older adults’ health needs has been explored in previous studies. Results show increased confidence among healthcare professionals in assessing functional status [94] and sensory impairments [59]. However, these studies do not address the holistic health needs perspective, as highlighted in our study.

Study results showed that the assessment tools added consistency and structure to assessment conversations, highlighted health needs often overlooked in current practice, and provided holistic insights into the simulated older adult’s health. Participants’ reflections on the use of assessment tools and their potential outcomes align with the second stage of Kolb’s Experiential Learning Cycle, Reflective Observation. Such reflections in debriefing can help learners identify strengths and knowledge gaps, deepen their understanding, and drive further skill development [53, 95], as well as foster improvement in both technical and non-technical skills [96, 97]. Reflections on systematic assessment practices are especially pertinent in the Norwegian context, where standardized tools are used infrequently, methods vary across municipalities, and many professionals rely on clinical judgment rather than validated tools for service allocation [34]. Insufficient competence and unstructured assessments can lead to unmet needs and adverse outcomes [10]. Clear guidelines and established tools are essential for the early detection of functional decline [10, 29]. The WHO highlights that systematic health needs assessments also improve equitable access to services and support healthy behaviors [3]. Findings from a recent review found that systematic geriatric assessments can identify unexpressed needs, predict falls, and detect age-related conditions [98], underscoring the need to strengthen competence in using holistic assessment tools and interpreting scores to tailor health-promoting measures for older adults living at home.

In our study, healthcare professionals appreciated the opportunity to collaborate interprofessionally during simulation, which supported natural dialogue with the simulated older adult and enabled discussion of health needs from diverse professional perspectives to tailor appropriate measures. This experience aligns with previous research indicating that interprofessional education in elder care should be both experiential and social. Such approaches optimize patient-centered outcomes, support collaborative practice [99], and foster holistic discussions that draw on diverse skills to address the complex health needs of older adults [100]. Simulation-based interprofessional training has been shown to enhance team performance [48], foster empathy for older and improve understanding of their health needs [55]. Furthermore, the importance of interprofessional collaboration during health needs assessments is highlighted, as it has been shown to improve assessment accuracy [22].

The results show that participants found the systematic health needs assessment somewhat extensive. Participants expressed a preference for shorter screening tools and, in some cases, questioned the necessity of using all included tools. There is no universally agreed-upon standard for the use of assessment tools. For example, Comprehensive Geriatric Assessment (CGA) is considered the gold standard [101]. The World Health Organization (WHO) recommends Integrated Care for Older People (ICOPE), which includes tools for person-centered assessment [3–5]. Brief geriatric screening tools have also been identified to detect unexpressed needs in older adults living at home, although evidence of their effectiveness in improving outcomes remains limited [98]. This underscores the need for research testing systematic, holistic assessments, as well as further studies to ensure adequate interprofessional assessment and care-planning competencies among healthcare professionals to maintain consistency in practice [102]. When participants discuss and reflect on the advantages and limitations of the systematic, holistic health needs assessment, the use of different tools, and consider ways to improve them, they are engaging in the third stage of Kolb’s learning cycle, Abstract Conceptualization [95]. In this stage, learners synthesize their concrete simulation experiences and reflections, draw general lessons and broader insights, and consider how these lessons can be applied beyond the immediate context [53, 95].

Participants in our study perceived the health needs assessment simulation as realistic and valuable, reporting that it enhanced their preparedness for real-life assessments. Previous research similarly report that realistic simulations promote positive learning outcomes, with participants appreciating simulation as an engaging, hands-on pedagogical approach [103], and that authentic scenarios enhance engagement, skill development [104–106] and knowledge retention [105, 107]. On the other hand, previous findings show that limited realism in scenario can impede learning [105]. For example, a reduction in fidelity, such as the use of content that does not mirror real practice, settings and equipment that lack realism, or scenarios that fail to evoke genuine emotional responses, can undermine engagement and diminish the learning experience [108]. Importantly, effective learning requires adequate preparation, alignment of roles and tasks with learners’ experience level, social interaction [109] and active engagement [110].

In our study, participants described uncertainty and nervousness before the simulation, but both participants having roles in the scenario and the observers reported positive learning outcomes in the debriefing. Similar findings have been reported by Dyrstad et al., where participants often felt nervous before simulation but ultimately found it rewarding, highlighting the importance of a safe and supportive learning environment [111]. The optimal approach for achieving learning outcomes in simulation remains unclear. Role-play scenarios can enhance knowledge, self-efficacy, and interprofessional collaboration similarly to standardized patient simulations [112]. Evidence on the effect of active versus observer roles is mixed. Some studies favor active role participation [109, 113, 114], which has been shown to enhance knowledge acquisition [112, 115]. Other studies report similar outcomes for both active and observer roles [116, 117], while another study reported even superior learning outcomes for observers [110]. However, observational learning becomes most meaningful when structured tools and active engagement are provided [66], supporting our use of collaboration script for observers [65, 66].

Overall, the study findings indicate that participants engaged in and learned from the simulation training to practice systematic health needs assessments and reflected on their experiences during the debriefing. However, a consideration that emerged was that participants continued to experience some uncertainties despite the training they had received. Especially, the practical elements of vision assessment were perceived as challenging, and the selection of appropriate measures given their limited prior knowledge and experience in this health function. Still, participants believed that additional practice would enhance their confidence and competence in conducting such assessments. Similarly, a previous study found that home healthcare professionals initially felt unqualified to assess vision despite its recognized importance [74]. However, with continued practice, structured vision assessments became both acceptable and feasible [118]. A recent review found that healthcare professionals often feel insufficiently prepared after training and seek further instruction in health needs assessment. Extended skill development through practice or mentorship has shown positive effects [19], underscoring that competence evolves through ongoing, iterative engagement [53, 119].

The reflections and findings from our study provide valuable insights for refining the health needs assessment and for designing future simulation-based training. Applying these insights aligns with the fourth stage of Kolb’s learning cycle, Active Experimentation, which involves using knowledge gained through the simulation training and reflective debriefing to guide future practice [50]. This stage emphasizes the iterative nature of learning, in which experience, reflection, and conceptualization support the ongoing development of competence in holistic health needs assessments [53, 95, 120].

### Methodological considerations and study limitations

Data from the debriefing phases were analyzed using qualitative reflexive thematic analysis [90]. Although qualitative analysis is inherently subjective, collaboration among all authors during coding and interpretation enriched the process by incorporating diverse perspectives, enhancing transparency, and mitigating bias. The researchers’ involvement in the simulation may also have influenced participants’ behaviors and reflections, making reflexivity essential throughout the analysis.

The simulation training was developed through collaboration between academics and practitioners. Although older adults were not directly involved in the development, the study was presented to an advisory board that included representatives of older adults, who provided feedback on the content and focus.

Healthcare professionals assumed roles as older adults and their next of kin, which provided insights into the assessment process from multiple perspectives. Standardized patients may have increased authenticity and consistency in the scenario [121]. The participants’ prior experience and preparation during the briefing enabled them to perform roles realistically, even in challenging scenarios such as portraying an 80-year-old adult. The use of sight-reduction glasses, a shawl, a blanket, and a home-like laboratory setting further enhanced realism.

The simulation training was divided into two sessions to cover all assessment tools. Although together they encompassed a holistic health needs assessment, such an assessment is ideally conducted in a single home visit in an older adult. The separation may have disrupted continuity, reducing the realism and flow of a holistic assessment. However, dividing the training ensured sufficient time to practice and reflect on all tools.

During debriefing, some participants reported uncertainty about conducting assessments with the tools and suggesting measures with confidence. This may reflect either the need for additional training or the natural process of skill acquisition. Familiarizing participants with the tools beforehand might have enhanced confidence, though it would have reduced the opportunity to capture their initial experiences. Our study had a relatively small purposive sample, but recruitment ensured that participants were interprofessional and had relevant assessment experience. Their expertise likely shaped engagement and reflections, yet was essential for generating meaningful insights. The outcomes provide a foundation for developing similar simulations, testing the approach in practice, and refining systematic, holistic health needs assessment tools, ultimately supporting health-promotion efforts for older adults living at home.

## Conclusion

Findings from this study illustrate that simulation training created opportunities for interprofessional teams to practice and reflect on conducting systematic, holistic health needs assessments for older adults living at home, supported by standardized tools. During the training, teams collaborated in the assessment process and communicated naturally with both the older adult and their next of kin. Participants perceived that the assessment facilitated a holistic understanding of the older adult’s health needs and enabled them to use the assessment scores to recommend tailored health-promotion measures. They found the training relevant and useful, with the scenario and setting perceived as authentic, although they also emphasized a need for ongoing practice to further strengthen their skills.

### Suggestions for future research

- Examine the feasibility and practical implications of conducting systematic, holistic assessments in real-world home healthcare contexts with older adults.
- Investigate the potential of conducting simulation training within participants’ workplaces to enhance realism, contextual relevance, and time efficiency.
- Pursue further work to develop and test more concise yet holistic assessment tools that balance thoroughness with feasibility in everyday practice.
- Explore strategies for implementing systematic and holistic assessments into routine home healthcare services, including organizational and professional considerations.

## Data Availability

The datasets generated and analyzed during this study are not publicly available owing to ethical considerations inherent in this research but are available from the corresponding author upon reasonable request.

## Abbreviations

ICOPE: Integrated Care for Older People
WHO: The World Health Organization
ADL: Activities of daily living
SPPB: Short Physical Performance Battery
CFS: Clinical Frailty Scale
UCLA: 3-Item Loneliness scale
AUDIT-4: Alcohol Use Disorders Identification Test
IPLOS: Norwegian Information System for the Nursing and Care Sector
SIKT: The Norwegian Agency for Shared Services in Education and Research
